# Work honored by Nobel prizes clusters heavily in a few scientific fields

**DOI:** 10.1371/journal.pone.0234612

**Published:** 2020-07-29

**Authors:** John P. A. Ioannidis, Ioana-Alina Cristea, Kevin W. Boyack

**Affiliations:** 1 The Meta-Research Innovation Center at Stanford (METRICS), Stanford, CA, United States of America; 2 The Meta-Research Innovation Center Berlin (METRIC-B), Berlin, Germany; 3 Department of Brain and Behavioral Science, University of Pavia, Pavia, Italy; 4 SciTech Strategies, Inc., Albuquerque, NM, United States of America; Universitat de Barcelona, SPAIN

## Abstract

We aimed to assess whether Nobel prizes (widely considered the most prestigious award in science) are clustering in work done in a few specific disciplines. We mapped the key Nobel prize-related publication of each laureate awarded the Nobel Prize in Medicine, Physics, and Chemistry (1995–2017). These key papers mapped in only narrow sub-regions of a 91,726-cluster map of science created from 63 million Scopus-indexed published items. For each key Nobel paper, a median of 435 (range 0 to 88383) other Scopus-indexed items were published within one year and were more heavily cited than the Nobel paper. Of the 114 high-level domains that science can be divided into, only 36 have had a Nobel prize. Five of the 114 domains (particle physics [14%], cell biology [12.1%], atomic physics [10.9%], neuroscience [10.1%], molecular chemistry [5.3%]) have the lion’s share, accounting in total for 52.4% of the Nobel prizes. Using a more granular classification with 849 sub-domains shows that only 71 of these sub-domains (8.3%) have at least one Nobel-related paper. Similar clustering was seen when we mapped all the 40,819 Scopus-indexed publications representing the career-long output of all the Nobel laureates. In conclusion, work resulting in Nobel prizes is concentrated in a small minority of scientific disciplines.

## Introduction

The Nobel prize is undoubtedly the most visible, influential, and coveted award across science. Nobel laureates represent a readily demarcated and most illustrious cohort of exceptional scientists, and data on their work have been repeatedly used [1–14] to assess aspects of citation impact, perception of rewards, credit allocation, paradigm shifts, and networking in science. Much can be learned by studying this influential cohort. A fundamental question is what disciplines of science are really covered by these top awards.

Not all fields of scholarship are thematically covered by the Nobel prizes, but the three prizes that focus on the natural and biomedical sciences (Chemistry, Physics, and Physiology/Medicine) have very wide coverage. In theory, almost any work within the broad range of natural sciences or biomedicine would be potentially eligible for this honor, if it has tremendous impact. However, a different viewpoint may be that these awards should be sharply focused in specific disciplines. Particular areas or sub-disciplines may attract far more Nobel honors than others and some investigators have suggested that a select scientific elite is more deserving and legitimately attract more honors, prizes; according to this view, a relatively constrained number of ideas and scholars push the boundaries of science [11]. Conversely, clustering in a few fields may herald bias and undeserved neglect of many/most scientific disciplines from these utmost rewards.

While it is difficult, if not impossible, to answer reliably whether any clustering is deserved or undeserved for this top recognition, at a minimum it would help to understand whether this clustering of favored scientific disciplines does exist and, if so, how strong it is. If it does exist, what fields are mostly preferred and how strong is the selection? How many major fields have never been honored by Nobel prizes? Here we aimed to address these issues systematically by mapping Nobel prize work in Physics, Chemistry, and Medicine/Physiology as well as the spread of the subsequent scientific publications that cited this work within a comprehensive map of science [15].

## Results

**Fig 1:**
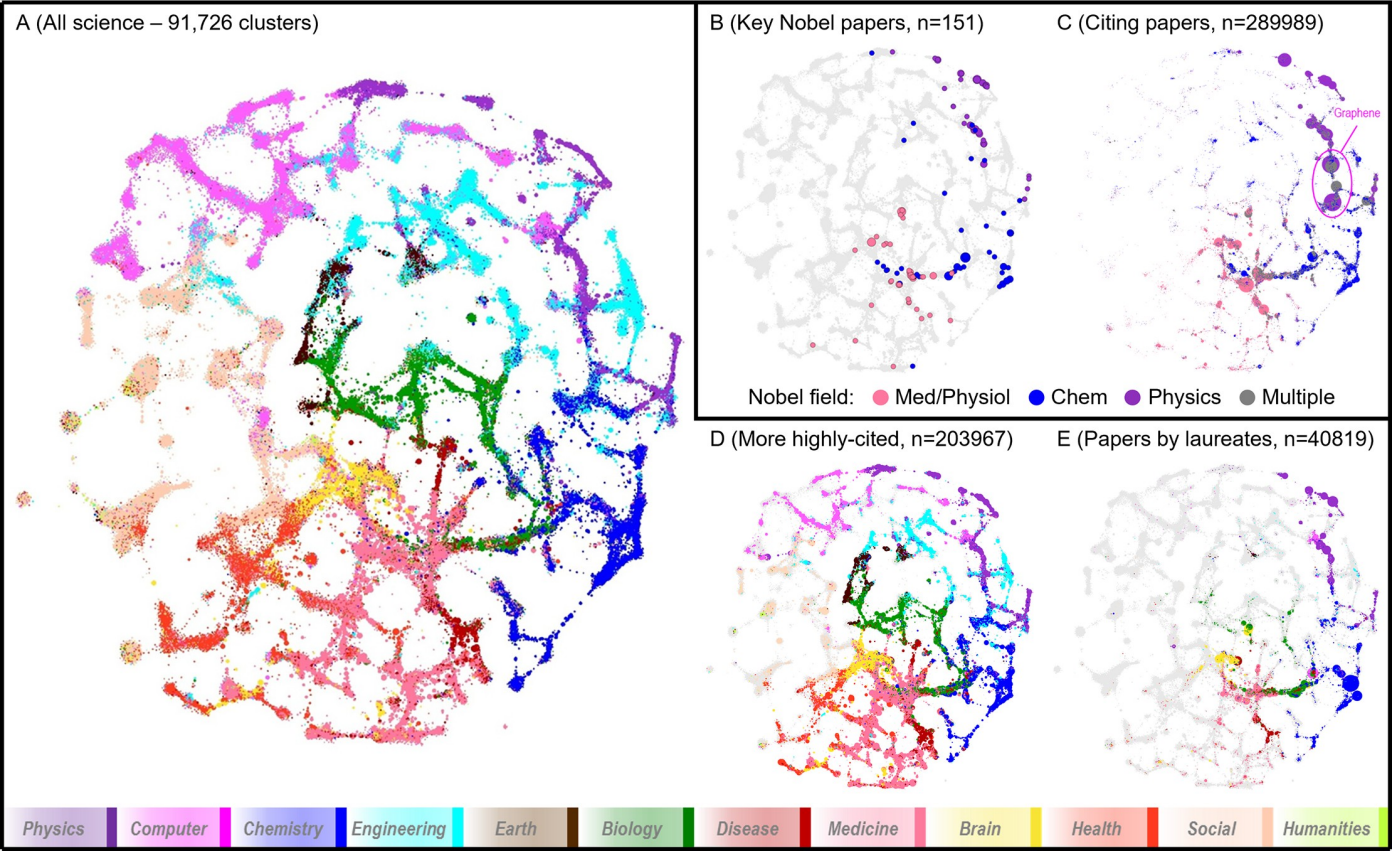
Location of papers in a map of science. (A) Map of science 91,726 clusters and associated large disciplines. The color legend is derived from the original construction of that map of science [15]). (B) The location of the key papers for Nobel prizes in Physics (purple), Chemistry (blue), and Physiology/Medicine (red) in 1995–2017. (C) The location of papers citing the key papers for Nobel prizes. Color legend as in panel B; grey color is used for areas that cite papers from two or more of three Nobel types. (D) The location of papers that have been more-cited than the respective Nobel key paper even though they were published within one calendar year of the Nobel key paper. Color legend as in panel A. (E) the location of the entire career-long publication record (40,819 papers) published by scientists awarded Nobel prizes in Physics, Chemistry, and Physiology/Medicine 1995–2017. Color legend as in panel A.

The map of science that we used (Fig 1) includes data from a total of 63 million published items, and it is divided into 12 major disciplines and a total of 91,726 clusters.

The full list of the key papers related to the Nobel work (with one paper selected per Nobel laureate) along with relevant data appear in the Supporting Information (S1 Table). Fig 1B shows where the key papers of the laureates are placed within the map of science: they tend to be localized to a small number of neighboring areas. Conversely, the vast majority of clusters in the science map contain no key Nobel papers in themselves or in their vicinity.

Fig 1C shows the localization in the science map of the papers that have cited the key Nobel papers as of May 2017. We examined the location of the papers that have cited the key Nobel papers, since an even spread of such papers across the science map could signal a wide interest of the research rewarded with the Nobel prize, while a more circumscribed and limited spread would suggest more topical interest. The vast majority of these citing papers are also in the same disciplinary areas as the key Nobel index papers. There is some modest spill over in some other, mostly neighboring, disciplines, but very few citing papers are in areas that are not in the immediate vicinity of the Nobel key papers, if not in the same exact sub-discipline.

Fig 1D shows the location of papers that have been more-cited than the respective Nobel key paper even though they were published within one calendar year of the Nobel key paper. Their distribution is very wide, and it covers almost all major fields of science. The distribution is almost as variegated as the distribution of all papers in Fig 1A. For each key Nobel paper, a median of 435 (range 0 to 88383) other Scopus-indexed items were published within one year and were more heavily cited than the Nobel paper as of May 2017. If one adds published items that are not indexed in Scopus but can still be cited by Scopus-indexed items (mostly these would be books), a median of 915 items (range 0 to 128049) published within one year of the respective key Nobel papers were more heavily cited than the Nobel paper as of May 2017. In fact, there is only one case where the key Nobel paper has been the most-cited than anything else published within one year across science (the paper in Science on graphene [16]). A large share of the items that are more heavily cited than the respective key Nobel paper belong actually in the same field as the Nobel paper. Some caution is needed because these are crude citation counts and different fields and subfields have different citation densities. However, most Nobel papers come from fields with quite high citation densities. The full distribution of the more highly-cited per field is shown in the Supporting Information (S2 Table).

In order to map the location of the key Nobel papers in specific domains that are more granular than the 12 large disciplines of science, but more concentrative than the 91,726 finely delineated clusters, we used the DC2 classification system which has been previously described [17] and which breaks science into 114 domains, aggregated from the 91,726 clusters. Table 1 shows that of the 114 domains, only 36 have been touched at all by Nobel prizes and have at least 0.333 points (i.e. representing one of three laureates awarded a prize in a single year); of those 36 domains only 20 have at least 1 full point. Five domains (particle physics [14%], cell biology [12.1%], atomic physics [10.9%], neuroscience [10.1%], molecular chemistry [5.3%]) have the lion’s share, accounting in total for 52.4% of the 69 Nobel prizes that we mapped, even though these 5 domains publish only 10.2% of all the Scopus-indexed papers. Using the DC3 classification [17], an intermediate level of aggregation that breaks science into 849 sub-domains, only 71 of these sub-domains (8.3%) are touched by at least one Nobel-related paper.

**Table 1:** The 36 domains that have been touched by at least 1 key paper from a Nobel prize*.

DC2	Domain	Major field	Papers in Scopus	Nobel points	Chemistry	Physics	Medicine
32	Particle Physics	6—Basic Physics	478415	9.667		9.667	
7	Cell Biology	5—Biochemistry	1085864	8.333	2.000		6.333
25	Atomic Physics	8—Appl Physics	576205	7.500	2.500	5.000	
8	Neuroscience	2—Medicine	952232	7.000	1.333		5.667
1	Molecular Chemistry	5—Biochemistry	1159261	3.667	3.667		
19	Molecular Biochemistry	5—Biochemistry	743948	2.667	1.333		1.333
15	Semiconductor Physics	8—Appl Physics	789874	2.667		2.667	
28	Magnetics	8—Appl Physics	573316	2.333		2.333	
38	Optoelectronics & Photonics	8—Appl Physics	546614	2.333	2.000	0.333	
64	Carbon Science	8—Appl Physics	293274	2.167	1.167	1.000	
106	Medical Imaging	2—Medicine	90218	1.833	0.833		1.000
70	Reproductive Medicine	2—Medicine	240215	1.667			1.667
37	Gastrointestinal Science	2—Medicine	495843	1.333	0.333		1.000
79	Receptor & Channel Science	5—Biochemistry	227776	1.333	1.333		
41	Nanochemistry	8—Appl Physics	454873	1.333	1.333		
11	Immunology	2—Medicine	921957	1.333			1.333
102	Nonlinear Dynamics	7—Artif Intell	110760	1.000	1.000		
10	Materials	9—Engineering	770157	1.000	1.000		
80	Sleep	2—Medicine	230643	1.000			1.000
48	Astronomy & Astrophysics	6—Basic Physics	253024	0.833		0.833	
23	Electrochemistry & Energy	8—Appl Physics	658303	0.833	0.833		
20	Mathematics	7—Artif Intell	560776	0.833	0.500	0.333	
110	Gasotransmitters	5—Biochemistry	64778	0.667			0.667
42	Tropical Disease & Parasites	3—Inf Disease	378898	0.667			0.667
31	Entomology	4—Sustainability	363741	0.667			0.667
63	AIDS	3—Inf Disease	284973	0.500			0.500
54	Climate Science	4—Sustainability	269801	0.500		0.500	
49	Cancer in Women	2—Medicine	349174	0.500			0.500
21	Ecology	4—Sustainability	447972	0.500	0.500		
91	Reconstructive Surgery	2—Medicine	156004	0.333			0.333
77	Analytical Chemistry	5—Biochemistry	217249	0.333	0.333		
68	Pharmacology	5—Biochemistry	259001	0.333	0.333		
52	Telecommunications	7—Artif Intell	348515	0.333		0.333	
30	Wildlife Science	4—Sustainability	384807	0.333	0.333		
18	Diabetes & Lifestyle/Prevention	2—Medicine	790664	0.333			0.333
16	Polymers	8—Appl Physics	657627	0.333	0.333		

*The following 76 fields did not have any key paper of any Nobel prize in Medicine/Physiology, Chemistry, or Physics in 1995–2017: Lithography, Offshore Mechanics, Tobacco, Acoustic Engineering, Electronic Packaging, Mycology, Petroleum Engineering, Brazil & Latin America, Information Science, Blood Disorders, Forestry, Law, Tuberculosis, Alcohol, Machining & Tribology, Philosophy, Plasma Physics, Energy Usage, Veterinary Sciences, Music & Sound, Mining Chemistry, Transportation, Bone Research, Energy Production, Separation Science, Nuclear Science, Cryptography, Metabolism Science, Environmental Engineering, Virology, Anesthesiology, Neurology & Neurosurgery, Endocrinology, Toxicology, Statistics, Emergency Medicine, Dermatology, Human Computing, Optical Materials, Archaeology, Pregnancy & Childbirth, Agricultural Policy, Urology, Composites, Nuclear Medicine, Ophthalmology, Planetary Science, Respiratory Diseases, Geological Engineering, Environmental Chemistry, Hematology, Civil Engineering, Liver Diseases, Operations Research, Dentistry, Power & Electricity, Microbiology, Brain/Vision & Hearing, Medicinal Chemistry, Oncology, Industrial Engineering, Fluid Mechanics, Animal Science, Networks, Cardiology, Orthopedics, Medieval Studies, Patient Care & Health Practitioners, Learning, Economics & Finance, Management, Computer Vision & Imaging

Computing, Marine Science, Geoscience, Plant Science, Psychiatry & Psychology, Governance

Of the 114 DC2 domains, only 20,10, and 15 domains are ever represented in Chemistry, Physics, and Medicine/Physiology Nobel prizes, respectively. Of note, Chemistry Nobel prizes are more scattered across more domains, many of which may not be seen as typical core chemistry areas (Table 1). The 76 domains that have not been touched by any Nobel prizes in Chemistry, Physics or Medicine/Physiology appear in the footnote of Table 1 and data on their numbering/classification in DC2 and the number of papers in each appear in the Supporting Information (S3 Table). One of the 76 is Economics and Finance, and certainly Nobel prizes in Economics (which we did not consider here) are likely to cluster in that domain. Some key papers of the same Nobel prize may also spill over in some additional fields. However, the majority of scientific fields seem to have been excluded by Nobel honors, even though in theory work done in these areas would be eligible for one or more of the three types of Nobel prizes that we analyzed.

One may argue that a single paper may not suffice to represent the work underlying a Nobel prize and a different rule may have selected different papers as being the most important hallmark of some Nobel-honored work. However, typically all the major papers underlying the same work tend to locate very close to each other in the map of science. A different empirical study [12] that tried to evaluate which authors are considered the ones deserving more credit in papers that led to Nobel recognitions selected papers with a different approach. Another group of scientists [13,14] used their own rules to arrive at a set of Nobel-related papers. These different selections largely pertain to the same domains as those papers that we selected. For example, 97 of the 144 papers that we selected overlap with the Li et al. selection [13,14] and the others are mostly in similar domains as those selected by Li et al. To take this a step further, we also identified all the 40,819 Scopus-indexed publications representing the career-long output of all the scientists awarded a Nobel prize in Physics, Chemistry, or Medicine/Physiology in 1995–2017. These 40,819 papers are mapped in Fig 1E and they still show strong clustering into the same, few scientific fields.

## Discussion

The present study shows that there is strong clustering of the work leading to Nobel awards in Medicine, Chemistry, and Physics. Five out of 114 fields of science (particle physics, atomic physics, cell biology, neuroscience, molecular chemistry) account for more than half of the awards. The majority of scientific fields were never honored with a Nobel prize in the examined period (1995–2017). This observation remains true regardless of the level of granularity in defining scientific fields and disciplines.

The current analysis also shows that almost always when a Nobel prize is awarded there are many other works that were published at the same time as the Nobel-honored work and that have been cited more extensively than the honored work. We should acknowledge, however, that citations do not cover the entire picture of potential impact. E.g., some work may lead to major advances but stop being cited because it helped the field move very far ahead. Conversely, a field where little progress is made may continue citing the same reference papers, e.g. nutritional epidemiology receives very high numbers of citations despite having made very little uncontested progress. Moreover, highly cited papers may differ substantially in ingenuity, innovation and translational potential [18]. Of course, not all highly-cited papers have identical value. Some types of articles and some books may attract a lot of citations, but there is no gold standard on measuring impact in ways that would be applicable to all science and would have high consensus across scientists doing different types of work (e.g. methods, discovery, applications, implementation). E.g. scientists may sometimes think more highly of the type of work that they do than other types of work.

Previous work [19] suggests that the most influential papers are extremely concentrated in few journals, especially in fields with high citation density. Nobel prizes also tend to come from fields with quite high citation densities. Thus clustering operates at multiple levels, including discipline, journal, as well as institutions [10]. Moreover, Nobel-awarded work may also influence future research, including allocation of funds and preferences of the most influential multidisciplinary journals regarding what to publish. This may reinforce a circle where privileged fields become even more privileged and achieve even more power and funds, relative to other scientific fields that remain more neglected. A similar circle may be reinforced at the level of what work will get recognized by Nobel awards in the following years.

One limitation of the present study is that it is sometimes difficult to identify a single paper that is clearly the epitome of the Nobel work. Other investigators [10] have used the same source of information as we did (information deposited in the Nobel official website) and tried to identify the most relevant papers for each laureate qualitatively by reading several papers that would have been candidates for selection. We preferred to use more objective bibliometric criteria of impact to select one paper for each laureate when there were several contesters, so as to avoid potential subjectivity. Regardless, the papers that we selected match substantially the corpus of papers selected by other authors [13,14]. Moreover, we also performed analyses focusing on the entire publication corpus of each laureate and this evaluation gave a similar overall picture, since most laureates (like most other scientists) largely focus their work in a particular single discipline.

Moreover, we should acknowledge that some disciplines may indeed not be very relevant for the three Nobel awards that we examined. For example, most work in law, philosophy, and medieval studies may be far too remote from medicine, chemistry, or physics. For several other disciplines, their exact level of relevance to the three Nobel awards may also be contested. Therefore, we do not argue that papers across all disciplines should be equally represented in Nobel awards. Moreover, we avoid testing the observed distribution of the past awards across disciplines versus some preconceived “gold standard” of what that distribution should ideally look like. Nevertheless, a perusal of the list of disciplines that have no Nobel award shows many disciplines that clearly would be relevant to the three Nobel award areas.

Even taking for granted that different disciplines unavoidably have different levels of relevance for Medicine/Physiology, Chemistry and Physics, the lack of representation of many fields in Nobel awards means that these fields never enjoy the prestige stemming from this most illustrious recognition. This may have implications for scientists and scientific communities, creating perceptions of second-class citizenship. Uneven prestige may affect institutional decisions in universities and research institutes on what work to cherish and support. Funders may also be influenced in their decisions on what to include or prioritize in their funding agenda. Even the perceptions of lay people on what fields of science are valuable (or even on what science is really dealing with) may be grossly distorted. One solution to achieve more evenness might be to create additional awards that are equally prestigious and that cover other fields besides those already primarily honored. For example, the addition of the Nobel Memorial Prize in Economic Sciences represents such an expansion of field coverage. It needs to be better appreciated that the chances of a scientist getting a Nobel prize are markedly different depending on what field he/she works in, regardless of the excellence, rigor or quality of his/her work. For example, 108,277 scientists have published at least 5 papers and focus primarily in Economics- and Business-related disciplines (Scopus data until end of 2017) [20], and there are 84 Nobel laureates in economics to-date. Thus, the odds of getting a Nobel prize is about 1 in 1,300 among economists. Conversely, there are over 3 million scientists working in medicine and life sciences and who have published at least 5 papers [20]. The average odds of getting a Nobel prize for them is miniscule compared with economists. Moreover, the odds of such a top recognition varies a lot for people working in different subdisciplines within biomedicine. In some subdisciplines, the odds of a scientist getting a Nobel prize may be better than 1:1,000, while in other subdisciplines no scientist may ever be honored, even though many tens of thousands of scientists work diligently in that subdiscipline.

It is unknown whether the clustering is driven by the nomination of candidates or by the selection of the winners among those who have been nominated, or both. One would need to assess whether strong clustering is already present among the group of nominees. Information on nominees is released however with a 50 years’ lag and thus the work of released nominees is largely outside the remit of the Scopus data.

Acknowledging these caveats, the clustering of Nobel-honored work in a few, restricted parts of the map of science seems highly prominent. The phenomenon may have different explanations, and different investigators may disagree on whether this intense clustering is desirable or not. Scientists working in the privileged domains may consider this inequality-in-honors highly appropriate, while other “excluded” scientists may consider it grossly unfair. There is a risk that this inequality creates a culture of exclusivity with some scientists considered second-class citizens simply because of the field in which they work or the type of research that they do. The clustering may reflect how different fields are perceived in general and/or by those scientists who nominate and award these prestigious prizes. A Matthew effect [21] may operate where the rich fields become richer, e.g. if the opinion of past awardees matters a lot either directly through their recommendations or by creating a frame upon which the next years’ prizes will be placed in context. This Matthew effect of reinforced field-specificity may apply also to any prestigious award, besides the Nobel prize. Some critics even claim [22] that the notion of trying to identify and single out one to three people goes against the communal spirit of science; for most major advances currently, there are important and distributed contributions across hundreds and thousands of researchers [23]. Fields that use more team science may thus be at a disadvantage and those where solitary investigators thrive may be at an advantage for a Nobel prize. Other investigators still propose that small teams are better suited for disruptive discoveries [24].

The large inequality in Nobel prize allocation across different domains of science may also resonate the debate about whether the relative extent to which scientific progress depends on bench science and reductionist discoveries, or more applied work, e.g. clinical or population research in the case of medicine [25,26]. Obviously, the Nobel prizes have focused to-date more on the former, with few prizes given for the latter (e.g. the work on *H*. *pylori* and peptic ulcer) [27]. Some may argue that we need to strengthen more bench discovery and this may create even greater concentration of honors to very few fields that are already privileged. Others may argue that we need to shift more attention to team work, since team work has become dominant in science [23]. Or to interdisciplinary work, data sciences, social determinants, applied clinical research, and other domains that may have even more translational value, but which have remained largely outside the remit of Nobel prizes [28,29]. We offer the data in this analysis as a stimulus for scientists in all fields, privileged or not, to ponder on these issues.

## Methods

### Map of science based on Scopus data

We used a previously developed comprehensive map of science which is based on the Scopus database [15]. The map consists of approximately 63 million documents through 2016 and was created in a multi-step process. The original map was created using 48 million documents through 2012 of which roughly half were Scopus indexed source documents from 1996–2012 and the other half were documents not indexed in Scopus but that were cited at least twice. 582 million direct citation links between the documents were used as the clustering input, and clustering was done using the smart local moving (SLM) modularity-based algorithm [30]. Clustering resulted in a four-level hierarchical solution with 91,726 clusters each containing at least 50 articles at the most granular (DC5, roughly 10^5^ clusters) level, 10,058 clusters at the DC4 (~10^4^ clusters) level, 849 sub-disciplines at the DC3 (~10^3^ clusters) level and 114 disciplines at the DC2 (~10^2^ clusters) level [17]. An additional 15 million papers through 2016 were later added to the existing clusters; each paper was added to the cluster to which it had the most reference links. Each paper resides in a cluster with other papers that either cite or are cited by them.

A visual map of the DC5 clusters (Fig 1A) was created using the following process: 1) similarity between pairs of clusters was calculated from the titles and abstracts of the documents in each topic using the BM25 text similarity measure [31], 2) the resulting similarity list was filtered to keep only the top-n (between 5 and 15) similarities per cluster, and 3) a layout of the clusters was generated using the DrL/OpenOrd algorithm [32], which gives each an x,y position based on the similarity graph. The DrL/OpenOrd algorithm is available at https://www.sandia.gov/smartin/software.html and is also available at https://www.cs.otago.ac.nz/homepages/smartin/software.php. Clusters with similar content are adjacent in the map. Each cluster has been designated as belonging primarily to one of twelve high-level fields and is colored accordingly. Color designations were made by assigning papers to fields using the UCSD journal-to-field assignments [33].

### Selection of key papers for Nobel laureates

We focus on Nobel prizes starting in 1995, since earlier prizes are more likely to recognize work done a long time ago and very old papers may not be contained in the Scopus database or may be less relevant to place in the map of science that we used based on Scopus data. We refer to the Nobel website (https://www.nobelprize.org/) for more details on the process of receiving nominations for the Nobel prize and choosing laureates.

We mapped the key Nobel prize-related publication of each laureate awarded the Nobel Prize between 1995 and 2017. We picked only a single paper per laureate, to avoid giving more weight to the work of some laureates over the others. Key papers (one per laureate) were identified from the Advanced Background Material/Advanced Information section for each Nobel Prize, as listed the official website (https://www.nobelprize.org/). As a general rule, both citation count and main authorship were considered in choosing a key paper for each author. Main authorship was defined as being listed as 1^st^ or last author. Specifically, for each Nobel laureate, we selected the most highly cited paper listed in the advanced information and where the laureate was a main author. Citation searches in Google Scholar were conducted between May 25th and July 18^th^, 2018. If a laureate also appeared on papers co-authored with other laureates, main authorship was prioritized (i.e., the paper where the 1^st^ laureate was 1^st^ or last was retained) unless the other paper (i.e., where the 1^st^ laureate was a not main author) had at least 3 times more citations.

A few special cases occurred. For a few laureates, no papers including them as main authors were present in the Advanced Information. In these cases, we selected the most highly cited paper that was present in the Advanced Information and had the laureate in any authorship position (i.e., even if not main). Precedence was given to papers listed in the Advanced Information, so as to minimize the need to have to decide whether other, not listed, papers, were relevant to the Nobel prize. In two cases, authorship on the most highly cited paper in the Advanced Information was determined alphabetically; the papers in question was included even if the laureates were not main authors, as the practice appeared to be the custom of the research group laureates belonged to. Conversely, if papers present in the Advanced Information section including the laureate as main author had low citation counts compared to others in the section that did not list the laureate as main author, citation counts were prioritized over main authorship if the difference was large (i.e., three-fold or more). For the years where there were very few papers listed in the Advanced Information section, where all had low citation counts or in which no Advanced Information document accompanied the Nobel Prize, precedence was given to the most highly-cited paper that seemed relevant for the prize.

For the analyses pertaining to the DC2 and DC3 aggregations, we allocated 1 point to each Nobel prize each year, which means for example that if there were three laureates with three different papers, each paper gets 0.333 points. With 23 years and three prizes for each, a total of 69 points could be allocated.

The analysis of citing papers considers all papers that cited the key Nobel papers and that had been indexed in Scopus as of May 2017. The same time stamp was used to search for papers and other published items (e.g. books) that had received more citations than each key Nobel paper even though they were published within one year of the respective key Nobel paper. Of course, competition for Nobel awards is not limited to single years (or three year windows), but the number of highly cited papers that are temporally congruent with each paper that led to an award places the citation impact of the honored work in better context, by adjusting for time since publication which is a key factor affecting citation counts.

### Sensitivity analyses

We performed similar analyses to understand the potential clustering, using the database of Nobel-related papers constructed by other authors [13,14]. We also mapped all 40,819 papers published by the Nobel laureates examined in this project.

## Supporting information

S1 Table Selected key papers for Nobel prizes in Physics, Chemistry, and Medicine/Physiology, 1995–2017. (DOCX)

S2 Table Number of papers in different fields published within one year of each Nobel paper that are more cited than the Nobel paper. (XLSX)

S3 Table 76 scientific domains that have had no key papers for the three types of Nobel prizes. (DOCX)
