# Supplementary material for: Work honored by Nobel prizes clusters heavily in a few scientific fields
Source: PLoS One. 2020 Jul 29;15(7):e0234612. doi: 10.1371/journal.pone.0234612 (PMC7390258; doi:10.1371/journal.pone.0234612)
Supplement: S1 Table — (DOCX) [file pone.0234612.s001.docx]

**S1 Table.** Selected key papers for Nobel prizes in Physics, Chemistry, and Medicine/Physiology, 1995-2017

| Laureate | Year | Paper | Citations (GS) |
| --- | --- | --- | --- |
| CHEMISTRY | | | |
| Crutzen, Paul J. | 1995 | Crutzen, P. J. & Andreae, M. O. Biomass burning in the tropics: impact on atmospheric chemistry and biogeochemical cycles. *Science (New York, N.Y.)* **250**, 1669-1678, doi:10.1126/science.250.4988.1669 (1990). | 2626 |
| Molina, Mario J. | 1995 | Molina, M. J. & Rowland, F. S. Stratospheric sink for chlorofluoromethanes: chlorine atom-catalysed destruction of ozone. *Nature* **249**, 810-812, doi:10.1038/249810a0 (1974). | 4700 |
| Rowland, F. Sherwood | 1995 | Molina, M. J. & Rowland, F. S. Stratospheric sink for chlorofluoromethanes: chlorine atom-catalysed destruction of ozone. *Nature* **249**, 810-812, doi:10.1038/249810a0 (1974). | 4700 |
| Smalley, Richard E. | 1996 | Kroto, H. W., Heath, J. R., O'Brien, S. C., Curl, R. F. & Smalley, R. E. C60: Buckminsterfullerene. *Nature* **318**, 162-163, doi:10.1038/318162a0 (1985). | 17577 |
| Kroto, Harold W | 1996 | Kroto, H. W., Heath, J. R., O'Brien, S. C., Curl, R. F. & Smalley, R. E. C60: Buckminsterfullerene. *Nature* **318**, 162-163, doi:10.1038/318162a0 (1985). | 17577 |
| Curl, Robert F. Jr. | 1996 | Curl, R. F. & Smalley, R. E. Probing c60. *Science (New York, N.Y.)* **242**, 1017-1022, doi:10.1126/science.242.4881.1017 (1988). | 631 |
| Boyer, Paul D. | 1997 | Boyer, P. D. The binding change mechanism for ATP synthase — Some probabilities and possibilities. *Biochimica et Biophysica Acta (BBA) - Bioenergetics* **1140**, 215-250, doi:https://doi.org/10.1016/0005-2728(93)90063-L (1993). | 1156 |
| Walker, John E. | 1997 | Walker, J. E., Saraste, M., Runswick, M. J. & Gay, N. J. Distantly related sequences in the alpha- and beta-subunits of ATP synthase, myosin, kinases and other ATP-requiring enzymes and a common nucleotide binding fold. *The EMBO journal* **1**, 945-951 (1982). | 5394 |
| Skou, Jens C. | 1997 | Skou, J. C. The influence of some cations on an adenosine triphosphatase from peripheral nerves. *Biochimica et Biophysica Acta* **23**, 394-401, doi:https://doi.org/10.1016/0006-3002(57)90343-8 (1957). | 2507 |
| Pople, John A. ^§^ | 1998 | Hariharan, P. C. & Pople, J. A. The influence of polarization functions on molecular orbital hydrogenation energies. *Theoretica chimica acta* **28**, 213-222, doi:10.1007/BF00533485 (1973) | 12909 |
| Kohn, Walter | 1998 | Kohn, W. & Sham, L. J. Self-Consistent Equations Including Exchange and Correlation Effects. *Physical Review* **140**, A1133-A1138, doi:10.1103/PhysRev.140.A1133 (1965). | 48353 |
| Zewail, Ahmed H.^§^ | 1999 | Zewail, A. H. Laser Femtochemistry. *Science (New York, N.Y.)* **242**, 1645-1653, doi:10.1126/science.242.4886.1645 (1988). | 755 |
| MacDiarmid, Alan G. | 2000 | Chiang, C. K. *et al.* Electrical Conductivity in Doped Polyacetylene. *Physical Review Letters* **39**, 1098-1101, doi:10.1103/PhysRevLett.39.1098 (1977). | 4117 |
| Heeger, Alan J. | 2000 | Shirakawa, H., Louis, E. J., MacDiarmid, A. G., Chiang, C. K. & Heeger, A. J. Synthesis of electrically conducting organic polymers: halogen derivatives of polyacetylene, (CH). *Journal of the Chemical Society, Chemical Communications*, 578-580, doi:10.1039/C39770000578 (1977). | 3701 |
| Shirakawa, Hideki | 2000 | Shirakawa, H., Louis, E. J., MacDiarmid, A. G., Chiang, C. K. & Heeger, A. J. Synthesis of electrically conducting organic polymers: halogen derivatives of polyacetylene, (CH). *Journal of the Chemical Society, Chemical Communications*, 578-580, doi:10.1039/C39770000578 (1977). | 3701 |
| Knowles, William S. | 2001 | Knowles, W. S. Asymmetric hydrogenation. *Accounts of Chemical Research* **16**, 106-112, doi:10.1021/ar00087a006 (1983). | 916 |
| Sharpless, K. Barry | 2001 | Katsuki, T. & Sharpless, K. B. The first practical method for asymmetric epoxidation. *Journal of the American Chemical Society* **102**, 5974-5976, doi:10.1021/ja00538a077 (1980). | 3188 |
| Noyori, Ryōji | 2001 | Miyashita, A. *et al.* Synthesis of 2,2'-bis(diphenylphosphino)-1,1'-binaphthyl (BINAP), an atropisomeric chiral bis(triaryl)phosphine, and its use in the rhodium(I)-catalyzed asymmetric hydrogenation of .alpha.-(acylamino)acrylic acids. *Journal of the American Chemical Society* **102**, 7932-7934, doi:10.1021/ja00547a020 (1980). | 1196 |
| Tanaka, Koichi | 2002 | Tanaka, K. *et al.* Protein and polymer analyses up to m/z 100 000 by laser ionization time-of-flight mass spectrometry. *Rapid Communications in Mass Spectrometry* **2**, 151-153, doi:10.1002/rcm.1290020802 (1988). | 3651 |
| Wüthrich, Kurt | 2002 | Pervushin, K., Riek, R., Wider, G. & Wüthrich, K. Attenuated T_2_ relaxation by mutual cancellation of dipole–dipole coupling and chemical shift anisotropy indicates an avenue to NMR structures of very large biological macromolecules in solution. *Proceedings of the National Academy of Sciences* **94**, 12366-12371, doi:10.1073/pnas.94.23.12366 (1997). | 2350 |
| Fenn, John B. | 2002 | Fenn, J. B., Mann, M., Meng, C. K., Wong, S. F. & Whitehouse, C. M. Electrospray Ionization for Mass Spectrometry of Large Biomolecules. *Science (New York, N.Y.)* **246**, 64-71 (1989). | 8537 |
| Agre, Peter | 2003 | Preston, G. M., Carroll, T. P., Guggino, W. B. & Agre, P. Appearance of Water Channels in *Xenopus* Oocytes Expressing Red Cell CHIP28 Protein. *Science (New York, N.Y.)* **256**, 385-387, doi:10.1126/science.256.5055.385 (1992). | 2092 |
| MacKinnon, Roderick | 2003 | Doyle, D. A. *et al.* The Structure of the Potassium Channel: Molecular Basis of K+ Conduction and Selectivity. *Science (New York, N.Y.)* **280**, 69-77, doi:10.1126/science.280.5360.69 (1998). | 6617 |
| Hershko, Avram | 2004 | Hershko, A., Heller, H., Elias, S. & Ciechanover, A. Components of ubiquitin-protein ligase system. Resolution, affinity purification, and role in protein breakdown. *The Journal of biological chemistry* **258**, 8206-8214 (1983). | 1101 |
| Ciechanover, Aaron | 2004 | Hershko, A., Heller, H., Elias, S. & Ciechanover, A. Components of ubiquitin-protein ligase system. Resolution, affinity purification, and role in protein breakdown. *The Journal of biological chemistry* **258**, 8206-8214 (1983). | 1101 |
| Rose, Irwin A. | 2004 | Hershko, A., Ciechanover, A., Heller, H., Haas, A. L. & Rose, I. A. Proposed role of ATP in protein breakdown: conjugation of protein with multiple chains of the polypeptide of ATP-dependent proteolysis. *Proceedings of the National Academy of Sciences of the United States of America* **77**, 1783-1786, doi:10.1073/pnas.77.4.1783 (1980). | 768 |
| Chauvin, Yves^§^ | 2005 | Chauvin, Y., Mussmann, L. & Olivier, H. A Novel Class of Versatile Solvents for Two-Phase Catalysis: Hydrogenation, Isomerization, and Hydroformylation of Alkenes Catalyzed by Rhodium Complexes in Liquid 1,3-Dialkylimidazolium Salts. *Angewandte Chemie International Edition in English* **34**, 2698-2700, doi:10.1002/anie.199526981 (1996). | 1996 |
| Grubbs, Robert H. | 2005 | Schwab, P., France, M. B., Ziller, J. W. & Grubbs, R. H. A Series of Well-Defined Metathesis Catalysts–Synthesis of [RuCl2(CHR′)(PR3)2] and Its Reactions. *Angewandte Chemie International Edition in English* **34**, 2039-2041, doi:10.1002/anie.199520391 (1995). | 1848 |
| Schrock, Richard R. | 2005 | Schrock, R. R. *et al.* Synthesis of molybdenum imido alkylidene complexes and some reactions involving acyclic olefins. *Journal of the American Chemical Society* **112**, 3875-3886, doi:10.1021/ja00166a023 (1990). | 1564 |
| Kornberg, Roger D. | 2006 | Kornberg, R. D. Chromatin Structure: A Repeating Unit of Histones and DNA. *Science (New York, N.Y.)* **184**, 868-871, doi:10.1126/science.184.4139.868 (1974). | 2365 |
| Ertl, Gerhard | 2007 | Jakubith, S., Rotermund, H. H., Engel, W., von Oertzen, A. & Ertl, G. Spatiotemporal concentration patterns in a surface reaction: Propagating and standing waves, rotating spirals, and turbulence. *Physical Review Letters* **65**, 3013-3016, doi:10.1103/PhysRevLett.65.3013 (1990). | 771 |
| Shimomura, Osamu | 2008 | Shimomura, O., Johnson, F. H. & Saiga, Y. Extraction, Purification and Properties of Aequorin, a Bioluminescent Protein from the Luminous Hydromedusan, Aequorea. *Journal of Cellular and Comparative Physiology* **59**, 223-239, doi:10.1002/jcp.1030590302 (1962). | 2616 |
| Chalfie, Martin | 2008 | Chalfie, M., Tu, Y., Euskirchen, G., Ward, W. & Prasher, D. Green fluorescent protein as a marker for gene expression. *Science (New York, N.Y.)* **263**, 802-805, doi:10.1126/science.8303295 (1994). | 8040 |
| Tsien, Roger Y. | 2008 | Heim, R., Cubitt, A. B. & Tsien, R. Y. Improved green fluorescence. *Nature* **373**, 663-664, doi:10.1038/373663b0 (1995). | 6450 |
| Ramakrishnan, Venkatraman | 2009 | Wimberly, B. T. *et al.* Structure of the 30S ribosomal subunit. *Nature* **407**, 327-339, doi:10.1038/35030006 (2000). | 2215 |
| Steitz, Thomas A. | 2009 | Ban, N., Nissen, P., Hansen, J., Moore, P. B. & Steitz, T. A. The complete atomic structure of the large ribosomal subunit at 2.4 A resolution. *Science (New York, N.Y.)* **289**, 905-920 (2000). | 3623 |
| Yonath, Ada E. | 2009 | Schluenzen, F. *et al.* Structure of Functionally Activated Small Ribosomal Subunit at 3.3 Å Resolution. *Cell* **102**, 615-623, doi:10.1016/S0092-8674(00)00084-2 (2000). | 1177 |
| Suzuki, Akira | 2010 | Miyaura, N., Yamada, K. & Suzuki, A. A new stereospecific cross-coupling by the palladium-catalyzed reaction of 1-alkenylboranes with 1-alkenyl or 1-alkynyl halides. *Tetrahedron Letters* **20**, 3437-3440, doi:https://doi.org/10.1016/S0040-4039(01)95429-2 (1979). | 1825 |
| Negishi, Elichi | 2010 | Negishi, E., King, A. O. & Okukado, N. Selective carbon-carbon bond formation via transition metal catalysis. 3. A highly selective synthesis of unsymmetrical biaryls and diarylmethanes by the nickel- or palladium-catalyzed reaction of aryl- and benzylzinc derivatives with aryl halides. *The Journal of Organic Chemistry* **42**, 1821-1823, doi:10.1021/jo00430a041 (1977). | 1052 |
| Heck, Richard F. | 2010 | Heck, R. F. & Nolley, J. P. Palladium-catalyzed vinylic hydrogen substitution reactions with aryl, benzyl, and styryl halides. *The Journal of Organic Chemistry* **37**, 2320-2322, doi:10.1021/jo00979a024 (1972). | 2272 |
| Shechtman, Dan | 2011 | Shechtman, D., Blech, I., Gratias, D. & Cahn, J. W. Metallic Phase with Long-Range Orientational Order and No Translational Symmetry. *Physical Review Letters* **53**, 1951-1953, doi:10.1103/PhysRevLett.53.1951 (1984). | 7314 |
| Lefkowitz, Robert J. | 2012 | De Lean, A., Stadel, J. M. & Lefkowitz, R. J. A ternary complex model explains the agonist-specific binding properties of the adenylate cyclase-coupled beta-adrenergic receptor. *The Journal of biological chemistry* **255**, 7108-7117 (1980). | 1667 |
| Kobilka, Brian K. | 2012 | Rasmussen, S. G. F. *et al.* Crystal structure of the human β2 adrenergic G-protein-coupled receptor. *Nature* **450**, 383, doi:10.1038/nature06325  https://www.nature.com/articles/nature06325#supplementary-information (2007). | 2036 |
| Karplus, Martin | 2013 | Field, M. J., Bash, P. A. & Karplus, M. A combined quantum mechanical and molecular mechanical potential for molecular dynamics simulations. *Journal of Computational Chemistry* **11**, 700-733, doi:10.1002/jcc.540110605 (1990). | 2277 |
| Levitt, Michael | 2013 | Warshel, A. & Levitt, M. Theoretical studies of enzymic reactions: Dielectric, electrostatic and steric stabilization of the carbonium ion in the reaction of lysozyme. *Journal of Molecular Biology* **103**, 227-249, doi:https://doi.org/10.1016/0022-2836(76)90311-9 (1976). | 3896 |
| Warshel, Arieh | 2013 | Warshel, A. & Levitt, M. Theoretical studies of enzymic reactions: Dielectric, electrostatic and steric stabilization of the carbonium ion in the reaction of lysozyme. *Journal of Molecular Biology* **103**, 227-249, doi:https://doi.org/10.1016/0022-2836(76)90311-9 (1976). | 3896 |
| Betzig, Eric | 2014 | Betzig, E. *et al.* Imaging Intracellular Fluorescent Proteins at Nanometer Resolution. *Science (New York, N.Y.)* **313**, 1642-1645, doi:10.1126/science.1127344 (2006). | 5519 |
| Hell, Stefan W. | 2014 | Hell, S. W. & Wichmann, J. Breaking the diffraction resolution limit by stimulated emission: stimulated-emission-depletion fluorescence microscopy. *Opt. Lett.* **19**, 780-782, doi:10.1364/OL.19.000780 (1994). | 3703 |
| Moerner, William E. | 2014 | Dickson, R. M., Cubitt, A. B., Tsien, R. Y. & Moerner, W. E. On/off blinking and switching behaviour of single molecules of green fluorescent protein. *Nature* **388**, 355-358, doi:10.1038/41048 (1997). | 1324 |
| Lindahl, Tomas | 2015 | Lindahl, T. Instability and decay of the primary structure of DNA. *Nature* **362**, 709-715, doi:10.1038/362709a0 (1993). | 5020 |
| Modrich, Paul | 2015 | Parsons, R. *et al.* Hypermutability and mismatch repair deficiency in RER^+^ tumor cells. *Cell* **75**, 1227-1236, doi:10.1016/0092-8674(93)90331-J (1993). | 1153 |
| Sancar, Aziz | 2015 | Sancar, A., Hack, A. M. & Rupp, W. D. Simple method for identification of plasmid-coded proteins. *Journal of bacteriology* **137**, 692-693 (1979). | 1043 |
| Sauvage, Jean-Pierre | 2016 | Dietrich-Buchecker, C. O. & Sauvage, J. P. Interlocking of molecular threads: from the statistical approach to the templated synthesis of catenands. *Chemical Reviews* **87**, 795-810, doi:10.1021/cr00080a007 (1987). | 716 |
| Sir Stoddard, J. Fraser | 2016 | Balzani, V., Credi, A., Raymo, F. M. & Stoddart, J. F. Artificial Molecular Machines. *Angewandte Chemie International Edition* **39**, 3348-3391, doi:10.1002/1521-3773(20001002)39:19<3348::Aid-anie3348>3.0.Co;2-x (2000). | 2269 |
| Feringa, Bernard L. | 2016 | Koumura, N., Zijlstra, R. W. J., van Delden, R. A., Harada, N. & Feringa, B. L. Light-driven monodirectional molecular rotor. *Nature* **401**, 152-155, doi:10.1038/43646 (1999). | 1340 |
| Dubochet, Jacques | 2017 | Dubochet, J. *et al.* Cryo-electron microscopy of vitrified specimens. *Quarterly reviews of biophysics* **21**, 129-228 (1988). | 1974 |
| Frank, Joachim | 2017 | Van Heel, M. & Frank, J. Use of multivariate statistics in analysing the images of biological macromolecules. *Ultramicroscopy* **6**, 187-194, doi:https://doi.org/10.1016/0304-3991(81)90059-0 (1981). | 650 |
| Henderson, Richard | 2017 | Henderson, R. *et al.* Model for the structure of bacteriorhodopsin based on high-resolution electron cryo-microscopy. *Journal of Molecular Biology* **213**, 899-929, doi: https://doi.org/10.1016/S0022-2836(05)80271-2 (1990). | 3205 |
| PHYSIOLOGY/MEDICINE | | | |
| Lewis, Edward B. | 1995 | Lewis, E. B. A gene complex controlling segmentation in Drosophila. *Nature* **276**, 565-570, doi:10.1038/276565a0 (1978). | 3564 |
| Nüsslein-Volhard, Christiane | 1995 | Nüsslein-Volhard, C. & Wieschaus, E. Mutations affecting segment number and polarity in Drosophila. *Nature* **287**, 795-801, doi:10.1038/287795a0 (1980). | 3979 |
| Wieschaus, Eric F. | 1995 | Nüsslein-Volhard, C. & Wieschaus, E. Mutations affecting segment number and polarity in Drosophila. *Nature* **287**, 795-801, doi:10.1038/287795a0 (1980). | 3979 |
| Doherty, Peter C. | 1996 | Zinkernagel, R. M. & Doherty, P. C. Restriction of in vitro T cell-mediated cytotoxicity in lymphocytic choriomeningitis within a syngeneic or semiallogeneic system. *Nature* **248**, 701-702, doi:10.1038/248701a0 (1974). | 2298 |
| Zinkernagel, Rolf M. | 1996 | Zinkernagel, R. M. & Doherty, P. C. Restriction of in vitro T cell-mediated cytotoxicity in lymphocytic choriomeningitis within a syngeneic or semiallogeneic system. *Nature* **248**, 701-702, doi:10.1038/248701a0 (1974). | 2298 |
| Prusiner, Stanley B. | 1997 | Prusiner, S. B. Novel proteinaceous infectious particles cause scrapie. *Science (New York, N.Y.)* **216**, 136-144 (1982). | 5019 |
| Furchgott, Robert F. | 1998 | Furchgott, R. F. & Zawadzki, J. V. The obligatory role of endothelial cells in the relaxation of arterial smooth muscle by acetylcholine. *Nature* **288**, 373-376 (1980). | 14540 |
| Ignarro, Louis J. | 1998 | Ignarro, L. J., Byrns, R. E., Buga, G. M. & Wood, K. S. Endothelium-derived relaxing factor from pulmonary artery and vein possesses pharmacologic and chemical properties identical to those of nitric oxide radical. *Circulation research* **61**, 866-879 (1987). | 5765 |
| Murad, Ferid | 1998 | Arnold, W. P., Mittal, C. K., Katsuki, S. & Murad, F. Nitric oxide activates guanylate cyclase and increases guanosine 3':5'-cyclic monophosphate levels in various tissue preparations. *Proceedings of the National Academy of Sciences of the United States of America* **74**, 3203-3207, doi:10.1073/pnas.74.8.3203 (1977). | 1527 |
| Blobel, Günter | 1999 | Blobel, G. & Dobberstein, B. Transfer of proteins across membranes. I. Presence of proteolytically processed and unprocessed nascent immunoglobulin light chains on membrane-bound ribosomes of murine myeloma. *The Journal of cell biology* **67**, 835-851, doi:10.1083/jcb.67.3.835 (1975). | 3393 |
| Carlsson, Arvid | 2000 | Carlsson, A. & Lindqvist, M. Effect of Chlorpromazine or Haloperidol on Formation of 3-Methoxytyramine and Normetanephrine in Mouse Brain. *Acta Pharmacologica et Toxicologica* **20**, 140-144, doi:10.1111/j.1600-0773.1963.tb01730.x (1963). | 2564 |
| Greengard, Paul | 2000 | Greengard, P., Valtorta, F., Czernik, A. J. & Benfenati, F. Synaptic vesicle phosphoproteins and regulation of synaptic function. *Science (New York, N.Y.)* **259**, 780-785 (1993). | 1278 |
| Kandel, Eric R. | 2000 | Kandel, E. R. & Schwartz, J. H. Molecular biology of learning: modulation of transmitter release. *Science (New York, N.Y.)* **218**, 433-443 (1982). | 1717 |
| Hartwell, Leland H. | 2001 | Hartwell, L. H. & Weinert, T. A. Checkpoints: controls that ensure the order of cell cycle events. *Science (New York, N.Y.)* **246**, 629-634 (1989). | 3313 |
| Hunt, Tim | 2001 | Evans, T., Rosenthal, E. T., Youngblom, J., Distel, D. & Hunt, T. Cyclin: a protein specified by maternal mRNA in sea urchin eggs that is destroyed at each cleavage division. *Cell* **33**, 389-396 (1983). | 1602 |
| Sir Nurse, Paul M. | 2001 | Gould, K. L. & Nurse, P. Tyrosine phosphorylation of the fission yeast cdc2+ protein kinase regulates entry into mitosis. *Nature* **342**, 39-45, doi:10.1038/342039a0 (1989). | 1296 |
| Brenner, Sydney | 2002 | Brenner, S. The genetics of Caenorhabditis elegans. *Genetics* **77**, 71-94 (1974). | 11719 |
| Horvitz, H. Robert | 2002 | Sulston, J. E. & Horvitz, H. R. Post-embryonic cell lineages of the nematode, Caenorhabditis elegans. *Developmental biology* **56**, 110-156 (1977). | 3180 |
| Sulston, John E. | 2002 | Sulston, J. E. & Horvitz, H. R. Post-embryonic cell lineages of the nematode, Caenorhabditis elegans. *Developmental biology* **56**, 110-156 (1977). | 3180 |
| Lauterbur, Paul C. | 2003 | Lauterbur, P. C. Image Formation by Induced Local Interactions: Examples Employing Nuclear Magnetic Resonance. *Nature* **242**, 190-191, doi:10.1038/242190a0 (1973). | 4482 |
| Sir Mansfield, Peter | 2003 | Mansfield, P. Multi-planar image formation using NMR spin echoes. *Journal of Physics C: Solid State Physics* **10**, L55-L58, doi:10.1088/0022-3719/10/3/004 (1977). | 2183 |
| Axel, Richard | 2004 | Buck, L. & Axel, R. A novel multigene family may encode odorant receptors: a molecular basis for odor recognition. *Cell* **65**, 175-187 (1991). | 4626 |
| Buck, Linda B. | 2004 | Buck, L. & Axel, R. A novel multigene family may encode odorant receptors: a molecular basis for odor recognition. *Cell* **65**, 175-187 (1991). | 4626 |
| Marshall, Barry J. | 2005 | Marshall, B. J. & Warren, J. R. Unidentified curved bacilli in the stomach of patients with gastritis and peptic ulceration. *Lancet (London, England)* **1**, 1311-1315 (1984). | 5925 |
| Warren, J. Robin | 2005 | Marshall, B. J. & Warren, J. R. Unidentified curved bacilli in the stomach of patients with gastritis and peptic ulceration. *Lancet (London, England)* **1**, 1311-1315 (1984). | 5925 |
| Fire, Andrew Z. | 2006 | Fire, A. *et al.* Potent and specific genetic interference by double-stranded RNA in Caenorhabditis elegans. *Nature* **391**, 806-811, doi:10.1038/35888 (1998). | 16725 |
| Mello, Craig C. | 2006 | Fire, A. *et al.* Potent and specific genetic interference by double-stranded RNA in Caenorhabditis elegans. *Nature* **391**, 806-811, doi:10.1038/35888 (1998). | 16725 |
| Capecchi, Mario R. | 2007 | Thomas, K. R. & Capecchi, M. R. Site-directed mutagenesis by gene targeting in mouse embryo-derived stem cells. *Cell* **51**, 503-512 (1987). | 2659 |
| Sir Evans, Martin J. | 2007 | Evans, M. J. & Kaufman, M. H. Establishment in culture of pluripotential cells from mouse embryos. *Nature* **292**, 154-156, doi:10.1038/292154a0 (1981). | 9208 |
| Smithies, Oliver | 2007 | Smithies, O., Gregg, R. G., Boggs, S. S., Koralewski, M. A. & Kucherlapati, R. S. Insertion of DNA sequences into the human chromosomal beta-globin locus by homologous recombination. *Nature* **317**, 230-234 (1985). | 1105 |
| Zur Hausen, Harald | 2008 | Durst, M., Gissmann, L., Ikenberg, H. & zur Hausen, H. A papillomavirus DNA from a cervical carcinoma and its prevalence in cancer biopsy samples from different geographic regions. *Proceedings of the National Academy of Sciences of the United States of America* **80**, 3812-3815, doi:10.1073/pnas.80.12.3812 (1983). | 2327 |
| Barré-Sinoussi, Françoise | 2008 | Barre-Sinoussi, F. *et al.* Isolation of a T-lymphotropic retrovirus from a patient at risk for acquired immune deficiency syndrome (AIDS). *Science (New York, N.Y.)* **220**, 868-871 (1983). | 8813 |
| Montaigner, Luc | 2008 | Barre-Sinoussi, F. *et al.* Isolation of a T-lymphotropic retrovirus from a patient at risk for acquired immune deficiency syndrome (AIDS). *Science (New York, N.Y.)* **220**, 868-871 (1983). | 8813 |
| Blackburn, Elizabeth H. | 2009 | Greider, C. W. & Blackburn, E. H. Identification of a specific telomere terminal transferase activity in Tetrahymena extracts. *Cell* **43**, 405-413 (1985). | 3368 |
| Greider, Carol W. | 2009 | Harley, C. B., Futcher, A. B. & Greider, C. W. Telomeres shorten during ageing of human fibroblasts. *Nature* **345**, 458-460, doi:10.1038/345458a0 (1990). | 5621 |
| Szostak, Jack W. | 2009 | Lundblad, V. & Szostak, J. W. A mutant with a defect in telomere elongation leads to senescence in yeast. *Cell* **57**, 633-643 (1989). | 970 |
| Edwards, Robert C. | 2010 | Steptoe, P. C. & Edwards, R. G. Birth after the reimplantation of a human embryo. *Lancet (London, England)* **2**, 366 (1978). | 2310 |
| Steinman, Ralph M. | 2011 | Steinman, R. M. & Cohn, Z. A. Identification of a novel cell type in peripheral lymphoid organs of mice. I. Morphology, quantitation, tissue distribution. *The Journal of experimental medicine* **137**, 1142-1162, doi:10.1084/jem.137.5.1142 (1973). | 2873 |
| Beutler, Bruce A. | 2011 | Poltorak, A. *et al.* Defective LPS signaling in C3H/HeJ and C57BL/10ScCr mice: mutations in Tlr4 gene. *Science (New York, N.Y.)* **282**, 2085-2088 (1998). | 7943 |
| Hoffmann, Jules A. | 2011 | Lemaitre, B., Nicolas, E., Michaut, L., Reichhart, J. M. & Hoffmann, J. A. The dorsoventral regulatory gene cassette spatzle/Toll/cactus controls the potent antifungal response in Drosophila adults. *Cell* **86**, 973-983 (1996). | 4210 |
| Sir Gurdon, John B. | 2012 | Gurdon, J. B. The developmental capacity of nuclei taken from intestinal epithelium cells of feeding tadpoles. *Journal of embryology and experimental morphology* **10**, 622-640 (1962). | 994 |
| Yamanaka, Shinya | 2012 | Takahashi, K. & Yamanaka, S. Induction of pluripotent stem cells from mouse embryonic and adult fibroblast cultures by defined factors. *Cell* **126**, 663-676, doi:10.1016/j.cell.2006.07.024 (2006). | 19615 |
| Rothman, James E. | 2013 | Sollner, T. *et al.* SNAP receptors implicated in vesicle targeting and fusion. *Nature* **362**, 318-324, doi:10.1038/362318a0 (1993). | 3166 |
| Schekman, Randy W. | 2013 | Novick, P., Field, C. & Schekman, R. Identification of 23 complementation groups required for post-translational events in the yeast secretory pathway. *Cell* **21**, 205-215, doi:10.1016/0092-8674(80)90128-2 (1980). | 1718 |
| Südhof, Thomas C. | 2013 | Geppert, M. *et al.* Synaptotagmin I: a major Ca2+ sensor for transmitter release at a central synapse. *Cell* **79**, 717-727 (1994). | 1385 |
| O’Keefe, John | 2014 | O'Keefe, J. & Dostrovsky, J. The hippocampus as a spatial map. Preliminary evidence from unit activity in the freely-moving rat. *Brain research* **34**, 171-175 (1971). | 4406 |
| Moser, May-Britt | 2014 | Fyhn, M., Molden, S., Witter, M. P., Moser, E. I. & Moser, M. B. Spatial representation in the entorhinal cortex. *Science (New York, N.Y.)* **305**, 1258-1264, doi:10.1126/science.1099901 (2004). | 970 |
| Moser, Edvard I. | 2014 | Hafting, T., Fyhn, M., Molden, S., Moser, M.-B. & Moser, E. I. Microstructure of a spatial map in the entorhinal cortex. *Nature* **436**, 801-806, doi:10.1038/nature03721 (2005). | 2261 |
| Youyou, Tu | 2015 | Tu, Y. Y. *et al.* [Studies on the constituents of Artemisia annua L. (author's transl)]. *Yao xue xue bao = Acta pharmaceutica Sinica* **16**, 366-370 (1981). | 90 |
| Campbell, William C. | 2015 | Egerton, J. R. *et al.* Avermectins, new family of potent anthelmintic agents: efficacy of the B1a component. *Antimicrobial agents and chemotherapy* **15**, 372-378, doi:10.1128/aac.15.3.372 (1979). | 376 |
| Ōmura, Satoshi | 2015 | Ikeda, H. *et al.* Complete genome sequence and comparative analysis of the industrial microorganism Streptomyces avermitilis. *Nature biotechnology* **21**, 526-531, doi:10.1038/nbt820 (2003). | 1165 |
| Ohsumui, Yoshinori | 2016 | Mizushima, N., Yamamoto, A., Matsui, M., Yoshimori, T. & Ohsumi, Y. In vivo analysis of autophagy in response to nutrient starvation using transgenic mice expressing a fluorescent autophagosome marker. *Molecular biology of the cell* **15**, 1101-1111, doi:10.1091/mbc.e03-09-0704 (2004). | 1875 |
| Hall, Jeffrey C. | 2017 | Rutila, J. E. *et al.* CYCLE is a second bHLH-PAS clock protein essential for circadian rhythmicity and transcription of Drosophila period and timeless. *Cell* **93**, 805-814 (1998). | 574 |
| Rosbash, Michael | 2017 | Hardin, P. E., Hall, J. C. & Rosbash, M. Feedback of the Drosophila period gene product on circadian cycling of its messenger RNA levels. *Nature* **343**, 536-540, doi:10.1038/343536a0 (1990). | 952 |
| Young, Michael W. | 2017 | Price, J. L. *et al.* *double-time* Is a *Novel Drosophila* Clock Gene that Regulates PERIOD Protein Accumulation. *Cell* **94**, 83-95, doi:10.1016/S0092-8674(00)81224-6 (1998). | 739 |
| PHYSICS | | | |
| Reines, Frederick | 1995 | Reines, F., Cowan, C. L., Harrison, F. B., McGuire, A. D. & Kruse, H. W. Detection of the Free Antineutrino. *Physical Review* **117**, 159-173, doi:10.1103/PhysRev.117.159 (1960). | 769 |
| Perl, Martin L. | 1995 | Perl, M. L. *et al.* Evidence for Anomalous Lepton Production in *e*^+_^ *e-* Annihilation. *Physical Review Letters* **35**, 1489-1492, doi:10.1103/PhysRevLett.35.1489 (1975). | 1736 |
| Lee, David M.^§^ | 1996 | Osheroff, D. D., Richardson, R. C. & Lee, D. M. Evidence for a New Phase of Solid **He**^3^. *Physical Review Letters* **28**, 885-888, doi:10.1103/PhysRevLett.28.885 (1972). | 609 |
| Osheroff, Douglas D. ^§^ | 1996 | Osheroff, D. D., Richardson, R. C. & Lee, D. M. Evidence for a New Phase of Solid **He**^3^. *Physical Review Letters* **28**, 885-888, doi:10.1103/PhysRevLett.28.885 (1972). | 609 |
| Richardson, Robert C. ^§^ | 1996 | Osheroff, D. D., Richardson, R. C. & Lee, D. M. Evidence for a New Phase of Solid **He**^3^. *Physical Review Letters* **28**, 885-888, doi:10.1103/PhysRevLett.28.885 (1972). | 609 |
| Chu, Steven^§^ | 1997 | Ashkin, A., Dziedzic, J. M., Bjorkholm, J. E. & Chu, S. Observation of a single-beam gradient force optical trap for dielectric particles. *Opt. Lett.* **11**, 288-290, doi:10.1364/OL.11.000288 (1986). | 6135 |
| Cohen-Tannoudji, Claude^§^ | 1997 | Dalibard, J. & Cohen-Tannoudji, C. Laser cooling below the Doppler limit by polarization gradients: simple theoretical models. *J. Opt. Soc. Am. B* **6**, 2023-2045, doi:10.1364/JOSAB.6.002023 (1989). | 1799 |
| Phillips, William D. ^§^ | 1997 | Phillips, W. D. & Metcalf, H. Laser Deceleration of an Atomic Beam. *Physical Review Letters* **48**, 596-599, doi:10.1103/PhysRevLett.48.596 (1982). | 945 |
| Laughlin, Robert B. | 1998 | Laughlin, R. B. Anomalous Quantum Hall Effect: An Incompressible Quantum Fluid with Fractionally Charged Excitations. *Physical Review Letters* **50**, 1395-1398, doi:10.1103/PhysRevLett.50.1395 (1983). | 5546 |
| Störmer, Horst L. | 1998 | Tsui, D. C., Stormer, H. L. & Gossard, A. C. Two-Dimensional Magnetotransport in the Extreme Quantum Limit. *Physical Review Letters* **48**, 1559-1562, doi:10.1103/PhysRevLett.48.1559 (1982). | 4466 |
| Tsui, Daniel C. | 1998 | Tsui, D. C., Stormer, H. L. & Gossard, A. C. Two-Dimensional Magnetotransport in the Extreme Quantum Limit. *Physical Review Letters* **48**, 1559-1562, doi:10.1103/PhysRevLett.48.1559 (1982). | 4466 |
| ‘t Hooft, Gerardus | 1999 | t Hooft, G. & Veltman, M. Regularization and renormalization of gauge fields. *Nuclear Physics B* **44**, 189-213, doi:https://doi.org/10.1016/0550-3213(72)90279-9 (1972). | 2652 |
| Veltman, Martinus J.C. | 1999 | t Hooft, G. & Veltman, M. Regularization and renormalization of gauge fields. *Nuclear Physics B* **44**, 189-213, doi:https://doi.org/10.1016/0550-3213(72)90279-9 (1972). | 2652 |
| Alferov, Zhores I.^§^ | 2000 | Kirstaedter, N. *et al.* Low threshold, large T/sub o/ injection laser emission from (InGa)As quantum dots. *Electronics Letters* **30**, 1416-1417, doi:10.1049/el:19940939 (1994). | 971 |
| Kroemer, H.^§^ | 2000 | Kroemer, H. Heterostructure bipolar transistors and integrated circuits. *Proceedings of the IEEE* **70**, 13-25, doi:10.1109/PROC.1982.12226 (1982). | 1207 |
| Kilby, Jack S. | 2000 | Kilby, J. S. Invention of the integrated circuit. *IEEE Transactions on Electron Devices* **23**, 648-654, doi:10.1109/T-ED.1976.18467 (1976). | 395 |
| Cornell, Eric A. | 2001 | Anderson, M. H., Ensher, J. R., Matthews, M. R., Wieman, C. E. & Cornell, E. A. Observation of Bose-Einstein Condensation in a Dilute Atomic Vapor. *Science (New York, N.Y.)* **269**, 198-201, doi:10.1126/science.269.5221.198 (1995). | 9142 |
| Ketterle, Wolfgang | 2001 | Davis, K. B. *et al.* Bose-Einstein Condensation in a Gas of Sodium Atoms. *Physical Review Letters* **75**, 3969-3973, doi:10.1103/PhysRevLett.75.3969 (1995). | 7284 |
| Wieman, Carl E. | 2001 | Cornish, S. L., Claussen, N. R., Roberts, J. L., Cornell, E. A. & Wieman, C. E. Stable 85Rb bose-einstein condensates with widely tunable interactions. *Phys Rev Lett* **85**, 1795-1798, doi:10.1103/PhysRevLett.85.1795 (2000). | 1189 |
| Davis Jr., Raymond | 2002 | Davis, R., Harmer, D. S. & Hoffman, K. C. Search for Neutrinos from the Sun. *Physical Review Letters* **20**, 1205-1209, doi:10.1103/PhysRevLett.20.1205 (1968). | 1910 |
| Koshiba, Masatoshi | 2002 | Fukuda, Y. *et al.* Evidence for Oscillation of Atmospheric Neutrinos. *Physical Review Letters* **81**, 1562-1567, doi:10.1103/PhysRevLett.81.1562 (1998). | 6407 |
| Giacconi, Riccardo | 2002 | Giacconi, R., Gursky, H., Paolini, F. R. & Rossi, B. B. Evidence for x Rays From Sources Outside the Solar System. *Physical Review Letters* **9**, 439-443, doi:10.1103/PhysRevLett.9.439 (1962). | 1266 |
| Abrikosov, Alexei A.^§^ | 2003 | Abrikosov, A. A. On the magnetic properties of superconductors of the second group. *Sov. Phys. JETP* **5**, 1174-1182 (1957). | 4734 |
| Ginzburg, Vitaly L. | 2003 | Ginzburg, V. L. On the theory of superconductivity. *Il Nuovo Cimento (1955-1965)* **2**, 1234-1250, doi:10.1007/bf02731579 (1955). | 4288 |
| Leggett, Anthony J. | 2003 | Leggett, A. J. A theoretical description of the new phases of liquid ^3^He. *Reviews of Modern Physics* **47**, 331-414, doi:10.1103/RevModPhys.47.331 (1975). | 2061 |
| Gross, David J. | 2004 | Gross, D. J. & Wilczek, F. Ultraviolet Behavior of Non-Abelian Gauge Theories. *Physical Review Letters* **30**, 1343-1346, doi:10.1103/PhysRevLett.30.1343 (1973). | 6065 |
| Politzer, H. David | 2004 | Politzer, H. D. Reliable Perturbative Results for Strong Interactions? *Physical Review Letters* **30**, 1346-1349, doi:10.1103/PhysRevLett.30.1346 (1973). | 5757 |
| Wilczek, Frank | 2004 | Gross, D. J. & Wilczek, F. Ultraviolet Behavior of Non-Abelian Gauge Theories. *Physical Review Letters* **30**, 1343-1346, doi:10.1103/PhysRevLett.30.1343 (1973). | 6065 |
| Glauber, Roy J. | 2005 | Glauber, R. J. Coherent and Incoherent States of the Radiation Field. *Physical Review* **131**, 2766-2788, doi:10.1103/PhysRev.131.2766 (1963). | 6469 |
| Hall, John L. | 2005 | Jones, D. J. *et al.* Carrier-Envelope Phase Control of Femtosecond Mode-Locked Lasers and Direct Optical Frequency Synthesis. *Science (New York, N.Y.)* **288**, 635-639, doi:10.1126/science.288.5466.635 (2000). | 3854 |
| Hänsch, Theodor W. | 2005 | Diddams, S. A. *et al.* Direct Link between Microwave and Optical Frequencies with a 300 THz Femtosecond Laser Comb. *Physical Review Letters* **84**, 5102-5105, doi:10.1103/PhysRevLett.84.5102 (2000). | 1261 |
| Mather, John C. | 2006 | Mather, J. C. *et al.* Measurement of the cosmic microwave background spectrum by the COBE FIRAS instrument. *The Astrophysical Journal* **420**, 439-444 (1994). | 1058 |
| Smoot, George F. | 2006 | Smoot, G. F. *et al.* Structure in the COBE differential microwave radiometer first-year maps. *The Astrophysical Journal* **396**, L1-L5 (1992). | 4154 |
| Fert, Albert | 2007 | Baibich, M. N. *et al.* Giant Magnetoresistance of (001)Fe/(001)Cr Magnetic Superlattices. *Physical Review Letters* **61**, 2472-2475, doi:10.1103/PhysRevLett.61.2472 (1988). | 10642 |
| Grünberg, Peter | 2007 | Grünberg, P., Schreiber, R., Pang, Y., Brodsky, M. & Sowers, H. Layered magnetic structures: Evidence for antiferromagnetic coupling of Fe layers across Cr interlayers. *Physical review letters* **57**, 2442 (1986). | 2591 |
| Nambu, Yoichiro | 2008 | Nambu, Y. & Jona-Lasinio, G. Dynamical Model of Elementary Particles Based on an Analogy with Superconductivity. I. *Physical Review* **122**, 345-358, doi:10.1103/PhysRev.122.345 (1961). | 6497 |
| Kobayashi, Makoto | 2008 | Kobayashi, M. & Maskawa, T. CP-Violation in the Renormalizable Theory of Weak Interaction. *Progress of Theoretical Physics* **49**, 652-657, doi:10.1143/ptp.49.652 (1973). | 11904 |
| Maskawa, Toshihide | 2008 | Kobayashi, M. & Maskawa, T. CP-Violation in the Renormalizable Theory of Weak Interaction. *Progress of Theoretical Physics* **49**, 652-657, doi:10.1143/ptp.49.652 (1973). | 11904 |
| Kuen Kao, Charles | 2009 | Kao, K. & Hockham, G. A. in *Proceedings of the Institution of Electrical Engineers.* 1151-1158 (IET). | 1135 |
| Boyle, Willard S. | 2009 | Boyle, W. S. & Smith, G. E. Charge coupled semiconductor devices. *Bell System Technical Journal* **49**, 587-593 (1970). | 1272 |
| Smith, George E. | 2009 | Boyle, W. S. & Smith, G. E. Charge coupled semiconductor devices. *Bell System Technical Journal* **49**, 587-593 (1970). | 1272 |
| Geim, Andre | 2010 | Novoselov, K. S. *et al.* Two-dimensional atomic crystals. *Proceedings of the National Academy of Sciences* **102**, 10451-10453 (2005). | 8273 |
| Novoselov, Konstantin | 2010 | Novoselov, K. S. *et al.* Electric field effect in atomically thin carbon films. *Science (New York, N.Y.)* **306**, 666-669 (2004). | 41428 |
| Perlmutter, Saul | 2011 | Perlmutter, S. *et al.* Measurements of Ω and Λ from 42 high-redshift supernovae. *The Astrophysical Journal* **517**, 565 (1999). | 16097 |
| Schmidt, Brian P. | 2011 | Riess, A. G. *et al.* Observational evidence from supernovae for an accelerating universe and a cosmological constant. *The Astronomical Journal* **116**, 1009 (1998). | 16597 |
| Riess, Adam G. | 2011 | Riess, A. G. *et al.* Observational evidence from supernovae for an accelerating universe and a cosmological constant. *The Astronomical Journal* **116**, 1009 (1998). | 16597 |
| Haroche, Serge | 2012 | Brune, M. *et al.* Observing the progressive decoherence of the “meter” in a quantum measurement. *Physical Review Letters* **77**, 4887 (1996). | 1839 |
| Wineland, David J. | 2012 | Monroe, C., Meekhof, D., King, B., Itano, W. M. & Wineland, D. J. Demonstration of a fundamental quantum logic gate. *Physical review letters* **75**, 4714 (1995). | 1987 |
| Englert, François | 2013 | Englert, F. & Brout, R. Broken symmetry and the mass of gauge vector mesons. *Physical Review Letters* **13**, 321 (1964). | 6369 |
| Higgs, Peter W. | 2013 | Higgs, P. W. Broken symmetries and the masses of gauge bosons. *Physical Review Letters* **13**, 508 (1964). | 7119 |
| Akasaki, Isamu | 2014 | Amano, H., Kito, M., Hiramatsu, K. & Akasaki, I. P-type conduction in Mg-doped GaN treated with low-energy electron beam irradiation (LEEBI). *Japanese Journal of Applied Physics* **28**, L2112 (1989). | 2326 |
| Amano, Hiroshi | 2014 | Amano, H., Sawaki, N., Akasaki, I. & Toyoda, Y. Metalorganic vapor phase epitaxial growth of a high quality GaN film using an AlN buffer layer. *Applied Physics Letters* **48**, 353-355 (1986). | 2628 |
| Nakamura, Shuji | 2014 | Nakamura, S., Mukai, T. & Senoh, M. Candela‐class high‐brightness InGaN/AlGaN double‐heterostructure blue‐light‐emitting diodes. *Applied Physics Letters* **64**, 1687-1689 (1994). | 4517 |
| Kajita, Takaaki | 2015 | Fukuda, Y. *et al.* Evidence for oscillation of atmospheric neutrinos. *Physical Review Letters* **81**, 1562 (1998). | 6385 |
| McDonald, Arthur B. | 2015 | Ahmad, Q. R. *et al.* Direct evidence for neutrino flavor transformation from neutral-current interactions in the Sudbury Neutrino Observatory. *Physical review letters* **89**, 011301 (2002). | 3305 |
| Thouless, David J. | 2016 | Kosterlitz, J. M. & Thouless, D. J. Ordering, metastability and phase transitions in two-dimensional systems. *Journal of Physics C: Solid State Physics* **6**, 1181 (1973). | 9941 |
| Haldane, F. Duncan M. | 2016 | Haldane, F. D. M. Nonlinear field theory of large-spin Heisenberg antiferromagnets: semiclassically quantized solitons of the one-dimensional easy-axis Néel state. *Physical Review Letters* **50**, 1153 (1983). | 3477 |
| Kosterlitz, J. Michael | 2016 | Kosterlitz, J. M. & Thouless, D. J. Ordering, metastability and phase transitions in two-dimensional systems. *Journal of Physics C: Solid State Physics* **6**, 1181 (1973). | 9941 |
| Weiss, Rainer | 2017 | Abbott, B. P. *et al.* Observation of gravitational waves from a binary black hole merger. *Physical review letters* **116**, 061102 (2016). | 4098 |
| Barish, Barry C. | 2017 | Abbott, B. P. *et al.* Observation of gravitational waves from a binary black hole merger. *Physical review letters* **116**, 061102 (2016). | 4098 |
| Thorne, Kip S. | 2017 | Abbott, B. P. *et al.* Observation of gravitational waves from a binary black hole merger. *Physical review letters* **116**, 061102 (2016). | 4098 |

*Note.* GS, Google Scholar; §, paper is not listed in the Nobel prize Additional background material/Advanced Information material
