# Supplementary material for: Work honored by Nobel prizes clusters heavily in a few scientific fields
Source: PLoS One. 2020 Jul 29;15(7):e0234612. doi: 10.1371/journal.pone.0234612 (PMC7390258; doi:10.1371/journal.pone.0234612)
Supplement: S3 Table — (DOCX) [file pone.0234612.s003.docx]

**S3 Table.** 76 scientific domains that have had no key papers for the three types of Nobel prizes

| **DC2** | **Domain** | **Large field** | **Papers in Scopus** |  |  |  |
| --- | --- | --- | --- | --- | --- | --- |
| 113 | Lithography | 8 - Appl Physics | 46115 |  |  |  |
| 112 | Offshore Mechanics | 9 - Engineering | 54015 |  |  |  |
| 111 | Tobacco | 2 - Medicine | 66899 |  |  |  |
| 109 | Acoustic Engineering | 9 - Engineering | 69116 |  |  |  |
| 108 | Electronic Packaging | 8 - Appl Physics | 72038 |  |  |  |
| 107 | Mycology | 3 - Inf Disease | 89136 |  |  |  |
| 105 | Petroleum Engineering | 9 - Engineering | 66714 |  |  |  |
| 104 | Brazil & Latin America | 4 - Sustainability | 56559 |  |  |  |
| 103 | Information Science | 1 - Civics | 91061 |  |  |  |
| 101 | Blood Disorders | 2 - Medicine | 115076 |  |  |  |
| 100 | Forestry | 4 - Sustainability | 81791 |  |  |  |
| 99 | Law | 1 - Civics | 64522 |  |  |  |
| 98 | Tuberculosis | 3 - Inf Disease | 133885 |  |  |  |
| 97 | Alcohol | 5 - Biochemistry | 125235 |  |  |  |
| 96 | Machining & Tribology | 9 - Engineering | 128828 |  |  |  |
| 95 | Philosophy | 1 - Civics | 90590 |  |  |  |
| 94 | Plasma Physics | 6 - Basic Physics | 134437 |  |  |  |
| 93 | Energy Usage | 9 - Engineering | 140412 |  |  |  |
| 92 | Veterinary Sciences | 3 - Inf Disease | 119516 |  |  |  |
| 90 | Music & Sound | 1 - Civics | 120846 |  |  |  |
| 89 | Mining Chemistry | 9 - Engineering | 122886 |  |  |  |
| 88 | Transportation | 9 - Engineering | 135476 |  |  |  |
| 87 | Bone Research | 2 - Medicine | 184511 |  |  |  |
| 86 | Energy Production | 9 - Engineering | 150062 |  |  |  |
| 85 | Separation Science | 9 - Engineering | 174889 |  |  |  |
| 84 | Nuclear Science | 9 - Engineering | 172648 |  |  |  |
| 83 | Cryptography | 7 - Artif Intell | 187651 |  |  |  |
| 82 | Metabolism Science | 5 - Biochemistry | 207566 |  |  |  |
| 81 | Environmental Engineering | 4 - Sustainability | 160932 |  |  |  |
| 78 | Virology | 3 - Inf Disease | 233911 |  |  |  |
| 76 | Anesthesiology | 2 - Medicine | 239086 |  |  |  |
| 75 | Neurology & Neurosurgery | 2 - Medicine | 244255 |  |  |  |
| 74 | Endocrinology | 2 - Medicine | 245389 |  |  |  |
| 73 | Toxicology | 5 - Biochemistry | 211968 |  |  |  |
| 72 | Statistics | 7 - Artif Intell | 211341 |  |  |  |
| 71 | Emergency Medicine | 2 - Medicine | 268253 |  |  |  |
| 69 | Dermatology | 2 - Medicine | 270134 |  |  |  |
| 67 | Human Computing | 7 - Artif Intell | 227732 |  |  |  |
| 66 | Optical Materials | 8 - Appl Physics | 227100 |  |  |  |
| 65 | Archaeology | 1 - Civics | 127740 |  |  |  |
| 62 | Pregnancy & Childbirth | 2 - Medicine | 293811 |  |  |  |
| 61 | Agricultural Policy | 4 - Sustainability | 218510 |  |  |  |
| 60 | Urology | 2 - Medicine | 312581 |  |  |  |
| 59 | Composites | 9 - Engineering | 266158 |  |  |  |
| 58 | Nuclear Medicine | 6 - Basic Physics | 275093 |  |  |  |
| 57 | Ophthalmology | 2 - Medicine | 321812 |  |  |  |
| 56 | Planetary Science | 6 - Basic Physics | 224056 |  |  |  |
| 55 | Respiratory Diseases | 2 - Medicine | 303716 |  |  |  |
| 53 | Geological Engineering | 9 - Engineering | 268704 |  |  |  |
| 51 | Environmental Chemistry | 5 - Biochemistry | 297245 |  |  |  |
| 50 | Hematology | 2 - Medicine | 347007 |  |  |  |
| 47 | Civil Engineering | 9 - Engineering | 311456 |  |  |  |
| 46 | Liver Diseases | 2 - Medicine | 382009 |  |  |  |
| 45 | Operations Research | 7 - Artif Intell | 350089 |  |  |  |
| 44 | Dentistry | 2 - Medicine | 411080 |  |  |  |
| 43 | Power & Electricity | 9 - Engineering | 439758 |  |  |  |
| 40 | Microbiology | 3 - Inf Disease | 461901 |  |  |  |
| 39 | Brain, Vision & Hearing | 1 - Civics | 455016 |  |  |  |
| 36 | Medicinal Chemistry | 5 - Biochemistry | 432630 |  |  |  |
| 35 | Oncology | 2 - Medicine | 571954 |  |  |  |
| 34 | Industrial Engineering | 7 - Artif Intell | 497747 |  |  |  |
| 33 | Fluid Mechanics | 9 - Engineering | 468439 |  |  |  |
| 29 | Animal Science | 4 - Sustainability | 458519 |  |  |  |
| 27 | Networks | 7 - Artif Intell | 584385 |  |  |  |
| 26 | Cardiology | 2 - Medicine | 678729 |  |  |  |
| 24 | Orthopedics | 2 - Medicine | 647520 |  |  |  |
| 22 | Medieval Studies | 1 - Civics | 259758 |  |  |  |
| 17 | Patient Care & Health Practitioners | 1 - Civics | 696756 |  |  |  |
| 14 | Learning | 1 - Civics | 517496 |  |  |  |
| 13 | Economics & Finance | 1 - Civics | 572754 |  |  |  |
| 12 | Management | 1 - Civics | 659537 |  |  |  |
| 9 | Computer Vision & Imaging | 7 - Artif Intell | 865071 |  |  |  |
| 6 | Computing | 7 - Artif Intell | 783879 |  |  |  |
| 5 | Marine Science | 4 - Sustainability | 659166 |  |  |  |
| 4 | Geoscience | 4 - Sustainability | 622071 |  |  |  |
| 3 | Plant Science | 4 - Sustainability | 728062 |  |  |  |
| 2 | Psychiatry & Psychology | 1 - Civics | 998464 |  |  |  |
| 0 | Governance | 1 - Civics | 867681 |  |  |  |
